# Application of Cleavable Linkers to Improve Therapeutic Index of Radioligand Therapies

**DOI:** 10.3390/molecules27154959

**Published:** 2022-08-04

**Authors:** Joseph Lau, Hwan Lee, Julie Rousseau, François Bénard, Kuo-Shyan Lin

**Affiliations:** 1 Department of Molecular Oncology, BC Cancer, Vancouver, BC V5Z 1L3, Canada; 2 Department of Radiology, University of Pennsylvania Perelman School of Medicine, Philadelphia, PA 19104, USA; 3 Department of Radiology, University of British Columbia, Vancouver, BC V5Z 1M9, Canada

**Keywords:** radioligand therapy, cleavable linker, brush border enzyme, kidney

## Abstract

Radioligand therapy (RLT) is an emergent drug class for cancer treatment. The dose administered to cancer patients is constrained by the radiation exposure to normal tissues to maintain an appropriate therapeutic index. When a radiopharmaceutical or its radiometabolite is retained in the kidneys, radiation dose deposition in the kidneys can become a dose-limiting factor. A good exemplar is [^177^Lu]Lu-DOTATATE, where patients receive a co-infusion of basic amino acids for nephroprotection. Besides peptides, there are other classes of targeting vectors like antibody fragments, antibody mimetics, peptidomimetics, and small molecules that clear through the renal pathway. In this review, we will review established and emerging strategies that can be used to mitigate radiation-induced nephrotoxicity, with a focus on the development and incorporation of cleavable linkers for radiopharmaceutical designs. Finally, we offer our perspectives on cleavable linkers for RLT, highlighting future areas of research that will help advance the technology.

## 1. Introduction

Radioligand therapy (RLT) is precision medicine mediated by radiopharmaceuticals to deliver radiation to cancer cells by targeting aberrant protein expression [1,2]. The ionizing radiation induces single- and double-strand DNA breaks in cancer cells to trigger mitotic catastrophe or apoptosis [3]. A therapeutic radiopharmaceutical is comprised of an antigen recognition molecule that binds to a target of interest, a radioisotope complex, and an optional linker that adjoins the two. With their inherent targeting properties, radiopharmaceuticals deliver high radiation doses to tumors while minimizing toxicity to normal tissues. RLT can be applied to hematological and solid malignancies and is particularly useful in oligometastatic settings. The clinical success of [^177^Lu]Lu-DOTATATE and [^177^Lu]Lu-PSMA-617 for the treatment of neuroendocrine tumors [4] and castration-resistant prostate cancers [5], respectively, has further bolstered the longstanding interest in RLT.

There are known limitations associated with RLT, such as the dependence of treatment efficacy on drug target expression. A predictable side effect of RLT is radiation damage to healthy tissues (e.g., bone marrow, kidneys, etc.) that occurs during the distribution and elimination phases for a radiopharmaceutical. Peptide and small molecule-based radiopharmaceuticals are typically cleared via the kidneys [6]. Nephrotoxicity is a potential concern if a radiopharmaceutical and/or its radiometabolite(s) becomes trapped within the tubular network of the parenchyma [7]. The distribution profile of a radiopharmaceutical in tumors and normal tissues dictates the probability of tumor control (TCP) and the risk of normal tissue complications (NTCP). The difference between TCP and NTCP is what defines the therapeutic index (TI) [8]. Early studies with ^177^Lu/^90^Y-labeled somatostatin analogues show that nephrotoxicity is a dose-limiting adverse event [9]. The current standard practice for RLT with peptide-based radiotherapeutics is to limit the activity dose given to patients to ensure that the maximum tolerable dose (MTD) for kidneys is not exceeded. This poses a challenge, as dose-limiting nephrotoxicity can lower the TI and prevent patients from receiving adequate quantities of radiation to achieve treatment responses. Per the FDA product label, the recommended dose for [^177^Lu]Lu-DOTATATE is 7.4 GBq every 8 weeks for 4 total doses in the presence of a renal protection regimen [10]. Developing strategies to reduce the renal retention of radiotherapeutics can have significant impacts on patient care and management.

In the present review, we will discuss radiation-induced nephrotoxicity and strategies employed in the clinic to mitigate damage related to NTCP, followed by the development and incorporation of cleavable linkers in emerging radiopharmaceutical designs.

## 2. Radioligand Therapy

RLT aims to deliver radioactivity to tumors and tumor-associated targets. This is achieved by attaching therapeutic radionuclides such as beta (β^−^) particle emitters or alpha (α) particle emitters to antigen recognition molecules (e.g., antibodies, antibody mimetics, peptides, peptidomimetics, small molecule inhibitors, etc.) for precision medicine [11]. RLT can sometimes be classified by the delivery vector (e.g., radioimmunotherapy [12]), the target choice (e.g., peptide receptor radionuclide therapy [13]), or the type of particle radiation (e.g., targeted alpha therapies [14]). Whereas conventional radiotherapeutic approaches are delivered externally, RLT is delivered systemically similar to chemotherapeutic agents [1]. Another distinguishing feature of RLT is that radiation is not uniformly delivered across cells [1]. Moreover, the tumor absorbed dose is dependent on multiple factors, including but not limited to, particle type, particle energy, range of emissions, tumor size, number of cells targeted, etc. [1]. RLT is administered in multiple doses over the course of weeks to allow for normal tissue recovery.

The most common type of particle used in RLT are β^−^ particles, which are electrons ejected from the nucleus of an atom during radioactive decay [15]. This is largely due to the fact that β^−^ emitters are widely accessible, and many emit photons that can be used for imaging [1]. β^−^ emitters have low linear energy transfer (LET; 0.2 keV/µM), which is the average energy deposited per unit track length along the track of an ionizing particle. The low LET leads to single-strand DNA breaks but delivers energy over longer distances (≤12 mm), which makes β^−^ emitters suitable for the treatment of solid tumors. The long range of β^−^ emitters potentiates a “crossfire” effect, where multiple cells can be targeted when only one binding event occurs [15]; however, this same attribute creates concerns of energy deposition beyond anatomical boundaries in the context of micrometastasis. Examples of FDA-approved radiotherapeutics that leverage β^−^ emitting radionuclides are [^177^Lu]Lu-DOTATATE [4] and [^177^Lu]Lu-PSMA-617 [16].

Besides β^−^ emitting radionuclides, α emitters have garnered significant clinical interest for RLT. α particles are helium nuclei that have high LET (80 keV/µM), but a short path range (50–100 µM) [11]. These properties make them ideal for targeting disseminated disease and micrometastases. α particles generate double-strand DNA breaks that are more difficult to repair than single-strand DNA breaks. The radiobiological effect of α particles is independent on cell cycle stage or oxygenation [17]. Compared with low energy X-rays, the relative biological effectiveness (RBE) of α emitters is 20 [18]. In a microdosimetric single cell model, 1 surface decay with an α particle is equivalent to approximately 1000 surface decays with a β^−^ emitter [19]. Favorable clinical responses have been observed with αemitters radiopharmaceuticals such as [^225^Ac]Ac-DOTATATE [20] and [^225^Ac]Ac-PSMA-617 [21] in patients who were refractory to ^177^Lu-treatment. While promising, it is important to remember that drug efficacy and drug toxicity are two sides of the same coin. Albeit mitigated by a shorter target range, α emitters carry a greater risk for toxicity.

## 3. Radiation-Induced Nephrotoxicity

The kidneys are critical organs for maintaining homeostasis, as they regulate extracellular fluid volume, fluid osmolality and acid-base balance, and remove toxins from blood. The filtration of a solute/molecule is dependent on size, charge, and plasma protein binding [7]. Molecules less than 12 kDa in MW (effective radius of 1.8 nm) can freely permeate through the glomerular basement membrane, while the filtration of molecules up to 70 kDa in MW (effective radius of 4.2 nm) is permissible but more restrictive [7]. Classically, positively charged or neutral molecules are known to be filtered more readily than negatively charged species, although this concept is not universally accepted [22,23]. Renal retention of radiopharmaceuticals is more of a complex phenomenon without a generalizable relationship to charge [24]. Except for antibody-based radiopharmaceuticals that are predominantly cleared through the hepatobiliary pathway, most of the radiopharmaceuticals used in RLT are eliminated through the kidneys.

After glomerular filtration, radiopharmaceuticals and their metabolites can be reabsorbed in the proximal tubules and subsequently retained. As articulated by Vegt et al., there are several mechanisms that can facilitate retention in the kidneys [7]. These include, but are not limited to, amino acid and oligopeptide transporters, megalin-cubilin mediated endocytosis, ligand-specific receptors, and peritubular absorption and tubular secretion [7]. Amino acid and oligopeptide transporters transport individual amino acids or short peptide units following cleavage of the parent peptide by brush border enzymes found on the surface of proximal tubular cells. Megalin and cubulin are endocytic receptors that transport a wide variety of proteins, peptides, and drugs. In knockout mice, the de Jong and Boerman groups demonstrated that megalin is essential for the reabsorption of radiolabeled peptides including somatostatin, exendin, neurotensin, and minigastrin derivatives [25,26]. As for ligand-specific receptors, there are oncological targets that are simultaneously expressed on renal cells (e.g., folate receptors and PSMA) [27]. In this scenario, it is more difficult to reduce renal exposure. For peritubular absorption and tubular secretion, active systems like the organic anion transporters and organic cationic transporters are involved in radiopharmaceutical retention [28].

Much of our understanding of radiation nephrotoxicity is derived from studies with external beam radiation therapy (EBRT), though the knowledge base is improving with respect to RLT. Radiation nephropathy generally takes months to manifest because of the slower turnover rate in cells compared with more active and proliferative tissues like the gastrointestinal tract or bone marrow. When renal function deteriorates, it can lead to proteinuria, azotemia, hypertension, anemia, and heart failure [6]. This is in addition to pathological changes like organ atrophy and inflammation, and scarring of the glomeruli and tubules [6]. Previously determined MTD for the kidneys based on EBRT was 23 Gy. This value has since been revised. According to the recent EANM guidelines for RLT for ^177^Lu-labeled PSMA-targeting ligands, the tolerance limit for kidneys is between 28–40 Gy [29]. The higher dose limit applies to patients without pre-existing risk factors (e.g., diabetes and old age) for renal disease. Given the possibility for co-morbidities and how cancer often afflicts the older demographic, it is important to find strategies to reduce the absorbed dose to kidneys.

## 4. Strategies to Mitigate Radiation-Induced Nephrotoxicity

**Figure 1:**
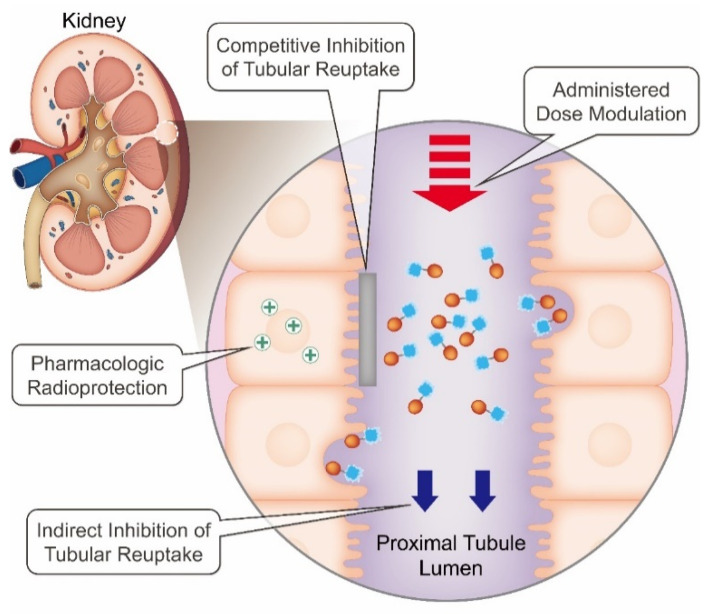
Strategies to reduce absorbed radiation dose to the kidneys.

Multiple strategies to reduce absorbed radiation dose to the kidneys exist, most of which have been examined in the setting of peptide receptor radionuclide therapy (PRRT) with radiolabeled somatostatin analogs such as [^177^Lu]Lu-DOTATATE. Administration of competitive renal tubular reuptake inhibitors is the most established method, while other methods include indirect interference of renal tubular reuptake, use of radioprotective agents, and personalized dosing (Figure 1). Besides the kidneys, high physiological uptake of [^177^Lu]Lu-DOTATATE can be observed in the spleen due to the expression of somatostatin receptors within the red pulp compartment [30,31]. Based on dosimetry estimations from the NETTER-1 trial [4,30], the bone marrow is considered the most critical organ as long as the kidneys are protected by co-administration of nephroprotective agents.

**Figure 2:**
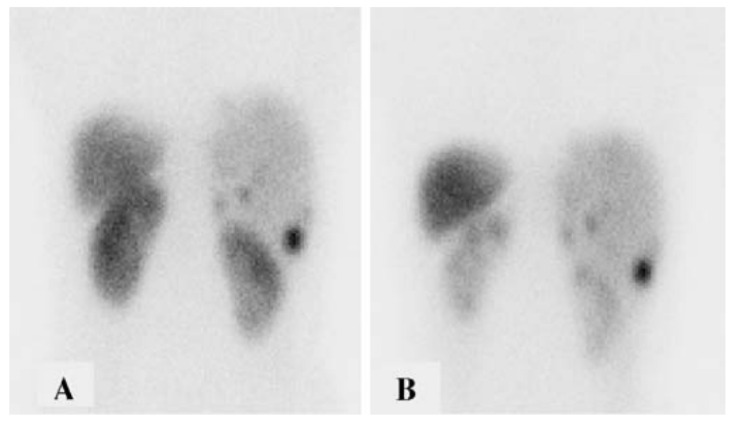
Abdominal scintigraphy in one patient after 220 MBq of [^111^In]In-DTPA^0^-octreotide. The images were obtained without (**A**) and with (**B**) co-infusion of 75 g of lysine. Renal radioactivity was 59% of control when lysine was infused. Reprinted with permission from ref. [32]. Copyright 2003 Springer-Verlag.

Reuptake of radiolabeled somatostatin analogs in the proximal tubule can be competitively inhibited with positively charged amino acids arginine and lysine [32] (Figure 2). Various amino acid mixtures and infusion protocols have been examined in the literature, with reduction in absorbed dose to the kidneys by 9–53% [32,33]. Given that the kidneys are major dose-limiting organs in PRRT [34], administration of an amino acid solution is now considered a required component of clinical PRRT protocols [34,35,36]. Unfortunately, amino acid infusion accounts for the most common acute side effects of PRRT, namely nausea and vomiting, which occur in 59% and 47% of patients, respectively, during the infusion [4]. Tolerability can be improved with the use of a compounded solution containing arginine and lysine, as well as administration of an antiemetic such as 5-HT_3_ antagonist [34]. When a compounded formulation is used with an antiemetic, the rates of nausea and vomiting are reduced to 31% and 14%, respectively [37], while the absorbed dose reduction in the kidneys is comparable at 33% [32].

Another competitive inhibitor of radiolabeled somatostatin analog reuptake at the proximal tubule is gelofusine, a plasma expander made of succinylated bovine gelatin that acts on the megalin-cubilin system [38]. Administration of gelofusine has been shown to reduce renal absorbed dose by 45%, which can potentially be further reduced with concurrent amino acid infusion [39,40]. However, its use has been limited due to concern for allergic reaction, where the rates of severe and all allergic reactions are estimated at 0.03–0.15% and 0.06–0.78%, respectively [41,42]. Currently, gelofusine is not routinely used for clinical PRRT except as an optional adjunct to amino acid infusion, and efforts to use albumin derivatives to minimize the risk of immunologic adverse events are at pre-clinical stages [43].

Several indirect strategies to decrease proximal tubule reuptake of somatostatin analogs have been described in pre-clinical studies. Renal uptake of [^111^In]In-octreotide could be reduced by colchicine, which interferes with endocytosis by inhibiting microtubule formation [44]. Depletion of the cellular energy required for endocytosis by inhibiting the citric acid cycle reduced renal [^111^In]In-octreotide uptake, but at the expense of significant renal toxicity [45]. Increasing the flow rate in the proximal tubule by the administration of fluid or use of the diuretics mannitol and acetazolamide did not result in reduced renal uptake, whereas furosemide paradoxically increased the renal uptake of [^111^In]In-DOTATOC by 44% [45,46]. In clinical PRRT, hydration is considered for patients with reduced baseline renal function, and diuretics only in cases of delayed renal outflow [35].

For a given radiation dose, radioprotective agents can be used to reduce radiation-induced nephrotoxicity. Amifostine is a prodrug that becomes activated by renal tubular cells to scavenge free radicals [47]. It is used clinically to mitigate cisplatin-induced nephrotoxicity [48], and it was shown to prevent PRRT-associated kidney injury in rats [49]. More recently, another radical scavenger, α1-microglobulin, was used to prevent PRRT-induced nephrotoxicity in mice [50]. The renin-angiotensin system has been implicated in the development of radiation-induced nephrotoxicity, and administration of the angiotensin-converting enzyme inhibitor, enalapril, for 3 months after PRRT mitigated renal toxicity in mice [51]. To date, radioprotective agents have not been clinically examined in the setting of PRRT.

The current PRRT guidelines recommend use of multiple treatment cycles at standardized dosages. However, the administered radionuclide dose can be modified based on dosimetry to minimize nephrotoxicity. Dose fractionation is routinely used in external beam radiotherapy to allow sublethal damage repair in normal tissues. This concept can be applied in PRRT with appropriate modifications [52], and dose fractionation has been shown to reduce nephrotoxicity [53]. It is feasible to perform personalized dosimetry to maximize the tumor absorbed dose while minimizing exposure to the kidneys [54].

## 5. Cleavable Linkers

**Figure 3:**
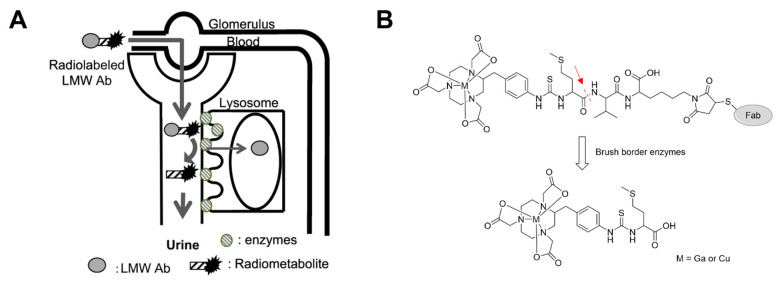
Renal brush border enzyme strategy. (**A**) The renal radioactivity levels of radiolabeled low molecular weight (LMW) Abs are reduced from early post-injection time by liberating a radiolabeled compound (radiometabolite) of urinary excretion from the parental LMW Abs by enzymes on the renal brush border membrane. Reprinted with permission from ref. [55]. Copyright 2021 Elsevier Inc. (**B**) Example of a cleavable linker that can be incorporated into radiopharmaceutical design. The scissile bond, shown in red, is cleaved by enzymes found on renal brush borders leading to the excretion of a radiometabolite.

In the previous section, we discussed various strategies to minimize radionuclide-induced nephrotoxicity using somatostatin analogs as exemplars. Some of these strategies are very effective and used as part of the standard-of-care in the administration of PRRT. However, pharmacokinetic issues like high renal exposure should ideally be addressed by structural optimization to improve clinical logistics. An emerging strategy to reduce renal retention of radiopharmaceuticals is the incorporation of a cleavable linker between the targeting ligand and the radiolabel complex [55]. The labile linker is designed to be recognized and cleaved by brush border enzymes (BBEs), such as neutral endopeptidase (also known as neprilysine), that are present on the membrane of proximal convoluted tubules [55]. Following cleavage, the radiometabolite is not retained in the kidneys, but is excreted into the urine to lower the radiation dose to the kidneys (Figure 3).

**Figure 4:**
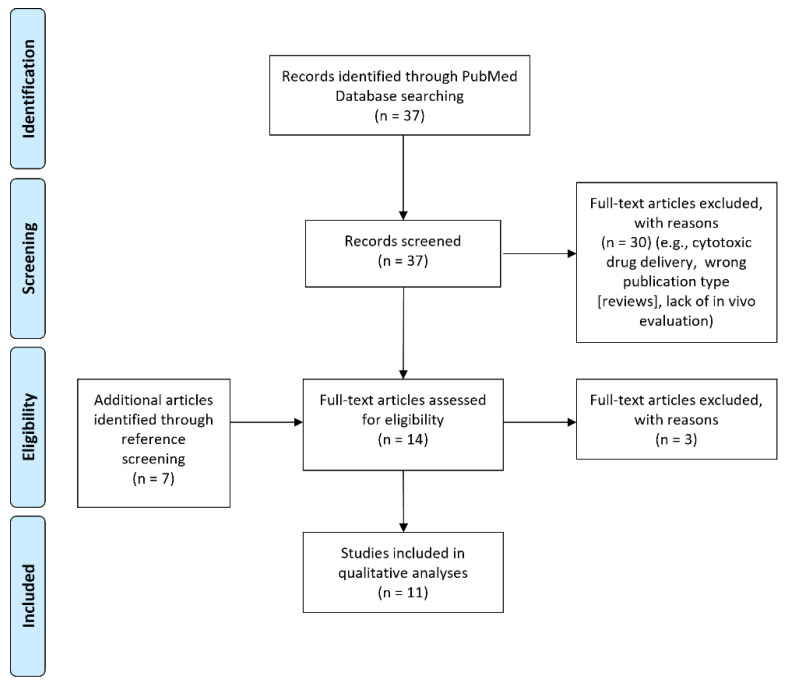
PRISMA flowchart showing study selection. PRISMA: Preferred Reporting Items for Systematic Reviews and Meta-analyses.

We conducted a search of the PubMed database using the following query: radiopharmaceuticals OR radiotherapy OR renal dose OR renal toxicity AND cleavable linkers. The abstracts of the potentially relevant articles were screened. References cited in the retrieved articles were subsequently searched to identify additional publications of interest that were not included in the initial search. Data was not filtered by date range. Figure 4 shows the flowchart of study selection, according to PRISMA criteria. Studies involving cleavable linkers applied to non-radiopharmaceuticals were excluded from analyses. The final qualitative analysis included 11 publications.

Arano et al. incorporated cleavable linkers to reduce the renal uptake retention of radiolabeled antibody fragments [56,57]. Fab fragments retain the target specificity of full-length IgGs, but are predominantly cleared via the renal pathway due to their smaller size. Thiolated Fab fragments were radiolabeled with 3′-[^131^I]iodohippuryl *N*^ε^-maleoyl-l-lysine ([^131^I]HML) or 3′-[^125^I]iodohippuryl *O*-((2-maleimidoethyl)carbamoyl)methyl-l-tyrosine ([^125^I]HMT). The glycyl-lysine sequence in HML and the glycyl-tyrosine sequence in HMT are substrates for BBEs. Selective cleavage of the glycyl-lysine or glycyl-tyrosine sequence led to the release of *meta*-iodohippuric acid. In a mouse model of osteosarcoma, [^131^I]HML-Fab showed approximately 75% lower radioactivity in kidneys compared with ^125^I-Fab that was directly radioiodinated at 3 h post-injection. More importantly, the tumor uptake was unaffected, which meant the tumor-to-kidney ratio was significantly improved.

Zhou et al. applied the glycyl-lysine linker to the design of an ^18^F-labeled single-domain antibody fragment, [^18^F]AlF-NOTA-Tz-TCO-GK-2Rs15d, raised against human growth factor receptor 2 (HER2) [58]. Cleavage by BBEs liberates the [^18^F]AlF-NOTA prosthetic group from the construct. Imaging and biodistribution studies were performed in mice bearing SKOV-3 ovarian cancer xenografts. Compared with [^125^I]SGMIB-2Rs15d, [^18^F]AlF-NOTA-Tz-TCO-GK-2Rs15d showed a 5-fold reduction in kidney uptake (10.5 ± 3.91 vs. 52.4 ± 11.6 %ID/g at 1 h). [^18^F]AlF-NOTA-Tz-TCO-GK-2Rs15d showed lower tumor uptake by 20% (3.46 ± 0.40 vs. 4.31 ± 0.52 %ID/g at 1 h). Because of the relative decreases in these organs, [^18^F]AlF-NOTA-Tz-TCO-GK-2Rs15d showed a 4-fold higher tumor-to-kidney ratio than [^125^I]SGMIB-2Rs15d. The biodistribution data for [^18^F]AlF-NOTA-Tz-TCO-2Rs15d (agent without the glycyl-lysine linker) was not available for comparison.

Vaidyanathan et al. synthesized PSMA-769, a prototypical prostate-specific membrane antigen (PSMA) inhibitor (((*S*)-1-carboxy-5-(4-iodobenzamido)pentyl)carbamoyl)glutamate with a glycyl-tyrosine cleavable linker [59]. PSMA-769 was radiolabeled with ^131^I or ^211^At. Biodistribution studies were performed in a transduced prostate cancer model that overexpresses PSMA. [^131^I]PSMA-769 had lower uptake in the kidneys at early time points compared to the control agent without the glycyl-tyrosine linker, but the difference was not statistically significant. There was a 6- to 7-fold lower kidney retention of [^131^I]PSMA-769 compared with the control at 21 h; however, the uptake of [^131^I]PSMA-769 in the tumor was concomitantly 3-fold lower. To demonstrate that the observation was BBE-mediated, the research group also synthesized [^125^I]PSMA-769-d-tyrosine to stabilize the glycyl-tyrosine linker. Urine analysis of mice receiving [^125^I]PSMA-769-d-tyrosine showed the unmetabolized intact molecule.

Yim et al. attempted to use a glycyl-lysine linker to reduce the renal retention of an exedin-4 derivative, [^64^Cu]Cu-NODAGA-*N*^ε^-maleoyl-l-lysyl-glycine (MAL)-exendin, for imaging glucagon-like peptide 1 receptor (GLP-1R) [60]. The radiopharmaceutical was evaluated in healthy rats. The incorporation of the cleavable sequence was ineffective. The biodistribution in tissues was similar for [^64^Cu]Cu-NODAGA-MAL-exendin-4 and the control [^64^Cu]Cu-NODAGA-exendin-4, with high kidney radioactivity levels noted at early and late timepoints (23.2 ± 8.06 vs. 25.2 ± 1.47 %ID/g at 1 h and 29.5 ± 3.64 vs. 22.1 ± 5.58 %ID/g at 24 h). This suggests that the type of radioprosthetic group can modulate enzyme accessibility and substrate recognition.

Building on the work performed by Barros and colleagues, which showed that the addition of a *C*-terminal lysine confers substrate specificity of Gly-Phe motif by neprilysin [61], the Arano group applied a tripeptide linker, Gly-Phe-Lys, to ^99m^Tc-labeled antibody fragments targeting c-kit [62]. ^99m^Tc was chelated using an isonicotinic acid derivative of 2-picolylglyine ([^99m^Tc]Tc-IPG). In tumor-bearing mice, [^99m^Tc]Tc-IPG-GFK-Fab showed similar tumor uptake as [^99m^Tc]Tc-IPG-GfK-Fab (7.65 ± 1.77 vs. 8.50 ± 1.54 %ID/g at 3 h), but significantly lower kidney uptake (3.74 ± 0.49 vs. 11.9 ± 2.20 %ID/g at 3 h). In vitro assays confirmed the mechanism of action. Liberation of [^99m^Tc]Tc-IPG-glycine was observed when [^99m^Tc]Tc-IPG-GFK(Boc) was incubated with brush border enzyme vesicles, while neither [^99m^Tc]Tc-IPG-GK(Boc) nor [^99m^Tc]Tc-IPG-GY(OMe) yielded the desired cleavage product.

**Figure 5:**
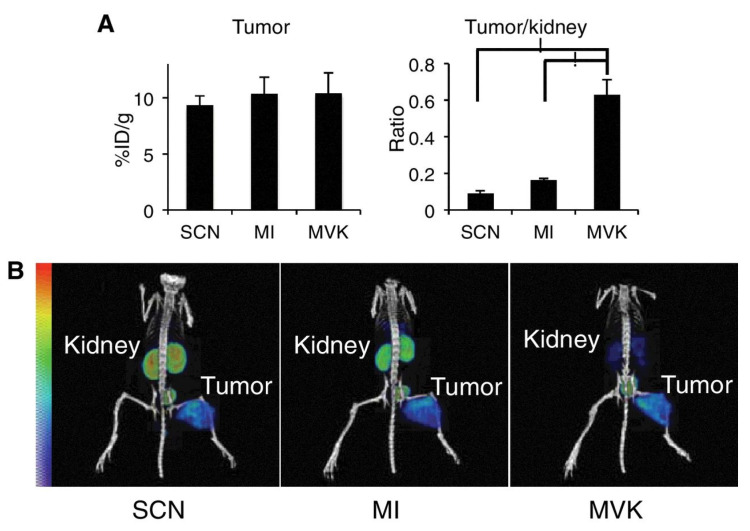
Tumor and kidney accumulation of radioactivity in mice after 3 h injection of ^67^Ga-labeled Fab fragments. (**A**) Radioactivity in tumor and corresponding tumor/kidney ratios after injection of ^67^Ga-NOTA-Fab (SCN), ^67^Ga-NOTA-MI-Fab (MI), and ^67^Ga-NOTA-MVK-Fab (MVK) in nude mice bearing SY tumors. Because of much lower renal radioactivity, ^67^Ga-NOTA-MVK-Fab yielded significantly higher tumor/kidney ratios than ^67^Ga-NOTA-MI-Fab and ^67^Ga-NOTA-Fab. (**B**) SPECT/CT images of nude mice. ^67^Ga-NOTA-MVK-Fab provided highest contrast tumor image. Reprinted with permission from ref. [63]. Copyright 2018 American Association for Cancer Research.

With the understanding that tripeptide linkages can reduce renal radioactivity levels with radiometal chelate complexes, other cleavable sequences were synthesized and evaluated. Uehara et al. utilized the Met-Val-Lys (MVK) sequence as a linker for a NOTA-conjugated ^67/68^Ga-labeled antibody fragment targeting c-kit [63]; MVK is cleaved by neprilysin between the Met-Val bond. The authors compared the MVK sequence with Met-Ile (MI) and a conventional thiourea linkage (Figure 5). Tumor uptake was similar for the three derivatives. The MI linker was able to reduce renal activity by approximately 33% compared with the isothiocyanate linker (64.1 ± 8.23 vs. 96.6 ± 8.46 %ID/g at 3 h), while the performance of the MVK linker was even better at 83% reduction (16.5 ± 1.64 %ID/g at 3 h). Consequently, the tumor-to-kidney ratio was the highest for the MVK derivative.

Research groups have leveraged the MVK motif to reduce renal activity for ^68^Ga-labeled NOTA-exendin 4 [64], ^68^Ga-labeled DOTA-PSMA inhibitor [65], and ^64^Cu/^67^Ga-labeled NOTA-Fab [66]. In each case, the addition of the cleavable linker was able to reduce the renal uptake of the tracer compared with the control. In the studies with the exendin 4 derivative and antibody fragment (targeting CD25), tumor uptake was unaffected. Compared with the original PSMA-targeting agent without a cleavable linker [67], tumor uptake was lower. This finding is likely the result of neprilysin overexpression on prostatic epithelial cells, which was previously reported with the LNCaP model [68]. The latter study highlights a potential limitation of the cleavable linker strategy, and the need to understand cancer biology for the design of radiopharmaceuticals.

**Figure 6:**
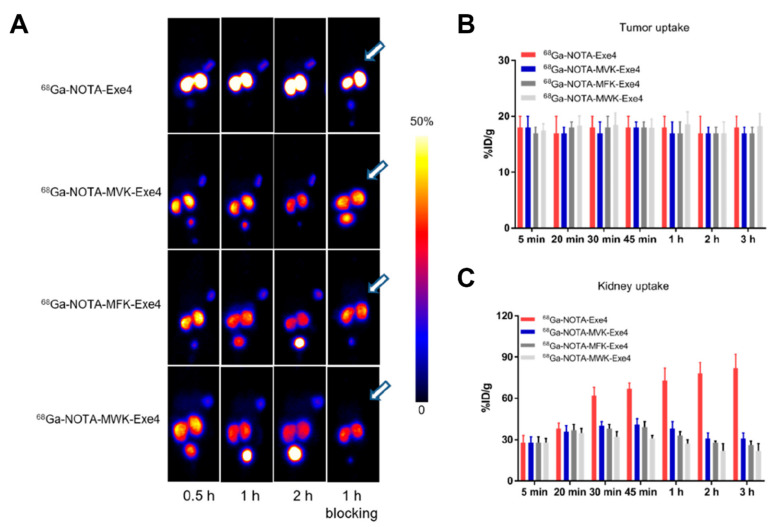
PET images of ^68^Ga-labeled exendin-4 derivatives (**A**). Quantification of kidney (**B**) and tumor (**C**) uptake in INS-1 tumor-bearing mice. Reprinted with permission from ref. [69]. Copyright 2021 American Chemical Society.

One of the more recent contributions to the field was a study performed by Zhang et al., where the authors used the exendin-4/GLP-1R system to compare MVK with two other sequences Met-Phe-Lys (MFK) and Met-Trp-Lys (MWK) [69]. Tumor uptake was similar in the INS-1 cancer model for the tested analogues (Figure 6). The MVK, MFK, and MWK linker successfully reduced renal activity accumulation by 40–55% compared with the control, with the MWK linker showing the best performance. Radio-HPLC analyses confirmed ^68^Ga-NOTA-Met-OH as the primary metabolite in kidneys and urine samples. The study shows that incorporation of a hydrophobic amino acid with a bulky side chain group is favourable for enhancing enzymolysis efficiency.

## 6. Perspective and Summary

There has been significant interest both academically and commercially to advance RLT for cancer therapy. For radiotherapeutics, patient dosing is constrained based on dosimetry to ensure that tolerance limits for normal tissues are not exceeded. Compared with radiolabeled antibodies that may confer significant hematological toxicity [70], the kidneys are often cited as potential dose-limiting organs for peptides and peptidomimetics, which have emerged as preferred choices for radiopharmaceutical development. Cleavable linkers act as pharmacokinetic modifiers to mitigate radiation-induced nephrotoxicity. It is easy to envision the use of cleavable linkers to potentiate RLT with other targeting vectors such as antibody fragments and antibody mimetics [71].

With platform technologies, it is important to identify and understand potential limitations. It is important that the cleavable linker remains stable against peptidases found in plasma and on the surfaces of cancer cells, otherwise payload delivery is negatively affected. Neprilysine, a key brush border enzyme for cleavable linkers, is expressed on other epithelial cancers such as non-small cell lung carcinoma [72] or ovarian carcinoma [73]. Depending on the relative expression, this may result in situations where tumor uptake decreases, negating the benefits of reduced renal activity. Some targeting vectors may be more amendable to the addition of a pharmacokinetic modifier than others. Inherently, the use of cleavable linkers will require careful optimization of the entire construct (e.g., linker sequence, position of the linker relative to the binder and radioprosthetic group, and the composition of the radioprosthetic group itself). The cleavable linker technology is limited to preclinical settings and for diagnostic applications. It will be important to ensure the translatability to radiotherapeutics, determine the absorbed dose instead of uptake at selected time points, and evaluate therapeutic efficacy.

Although it is still early days for cleavable linkers, the preclinical data achieved to date show good promise. With the iterative development of radiopharmaceuticals, we anticipate that the cleavable linker technology will continue to mature and be a part of the technical repertoire to improve patient outcomes and safety.

## Data Availability

Not applicable.
